# Deficiency of Carbonic Anhydrase II Results in a Urinary Concentrating Defect

**DOI:** 10.3389/fphys.2017.01108

**Published:** 2018-01-05

**Authors:** Devishree Krishnan, Wanling Pan, Megan R. Beggs, Francesco Trepiccione, Régine Chambrey, Dominique Eladari, Emmanuelle Cordat, Henrik Dimke, R. Todd Alexander

**Affiliations:** ^1^Department of Physiology, University of Alberta, Edmonton, AB, Canada; ^2^The Women's and Children's Health Research Institute, Edmonton, AB, Canada; ^3^Department of Cardio-Thoracic and Respiratory Science, University of Campania “Luigi Vanvitelli”, Naples, Italy; ^4^Institut National de la Santé et de la Recherche Médicale Diabète athérothrombose Thérapies Réunion Océan Indien (DéTROI) Université de La Réunion, CYROI, La Réunion, France; ^5^Centre National de la Recherche Scientifique, Délégation Paris Michel-Ange, Sainte-Clotilde, France; ^6^Service d'Explorations Fonctionnelles Rénales, Hôpital Felix Guyon, Centre Hospitalier Universitaire de la Réunion, La Réunion, France; ^7^Department of Cardiovascular and Renal Research, Institute of Molecular Medicine, University of Southern Denmark, Odense, Denmark; ^8^Department of Pediatrics, University of Alberta, Edmonton, AB, Canada

**Keywords:** carbonic anhydrase II, polyuria, urine concentration, aquaporin-1, metabolic

## Abstract

Carbonic anhydrase II (CAII) is expressed along the nephron where it interacts with a number of transport proteins augmenting their activity. Aquaporin-1 (AQP1) interacts with CAII to increase water flux through the water channel. Both CAII and aquaporin-1 are expressed in the thin descending limb (TDL); however, the physiological role of a CAII-AQP1 interaction in this nephron segment is not known. To determine if CAII was required for urinary concentration, we studied water handling in CAII-deficient mice. CAII-deficient mice demonstrate polyuria and polydipsia as well as an alkaline urine and bicarbonaturia, consistent with a type III renal tubular acidosis. Natriuresis and hypercalciuria cause polyuria, however, CAII-deficient mice did not have increased urinary sodium nor calcium excretion. Further examination revealed dilute urine in the CAII-deficient mice. Urinary concentration remained reduced in CAII-deficient mice relative to wild-type animals even after water deprivation. The renal expression and localization by light microscopy of NKCC2 and aquaporin-2 was not altered. However, CAII-deficient mice had increased renal AQP1 expression. CAII associates with and increases water flux through aquaporin-1. Water flux through aquaporin-1 in the TDL of the loop of Henle is essential to the concentration of urine, as this is required to generate a concentrated medullary interstitium. We therefore measured cortical and medullary interstitial concentration in wild-type and CAII-deficient mice. Mice lacking CAII had equivalent cortical interstitial osmolarity to wild-type mice: however, they had reduced medullary interstitial osmolarity. We propose therefore that reduced water flux through aquaporin-1 in the TDL in the absence of CAII prevents the generation of a maximally concentrated medullary interstitium. This, in turn, limits urinary concentration in CAII deficient mice.

## Introduction

Carbonic anhydrases are a group of zinc metalloenzymes that catalyze the reversible hydration of carbon dioxide into carbonic acid, which in solution rapidly dissociates into bicarbonate and a proton. The second isoform, carbonic anhydrase II (CAII) is soluble and is expressed in most tissues including red blood cells, osteoclasts, and the kidney (Spicer et al., 1979; Lewis et al., 1988). CAII is expressed to varying levels along the nephron with the exception of the thin ascending limb (Purkerson and Schwartz, 2007). It is most prominently expressed in intercalated cells of the distal nephron where it facilitates the secretion of protons (Schwartz, 2002), via a physical and functional interaction with the anion exchanger 1 (AE1). This interaction has been dubbed a transport metabolon (Vince and Reithmeier, 1998, 2000). Similarly in the proximal tubule CAII likely participates in sodium reabsorption from the filtrate by binding to apically expressed NHE3 and the basolaterally expressed NBCe1, increasing their activity via the provision of substrate (Becker and Deitmer, 2007; Krishnan et al., 2015).

CAII also physically and functionally interacts with aquaporin-1 (AQP1) (Vilas et al., 2013). It binds to a similar motif to that found in AE1, DADD, increasing the permeability of AQP1 to water (Vince and Reithmeier, 2000; Vilas et al., 2013). AQP1 is expressed in both the apical and basolateral membranes of the proximal tubule as well as the thin descending limb (TDL) of long looped nephrons (Nielsen et al., 1993b; Zhai et al., 2007). Both segments contribute significantly to AQP1 mediated water reabsorption, however, AQP1 mediated water flux across the TDL is also necessary for the concentration of urine (Chou et al., 1999). Although the role of AQP1 in the TDL is clear, to the best of our knowledge, the role of CAII in this segment is unknown.

Our observation that CAII augments water flux through AQP1 (Vilas et al., 2015) led us to posit that CAII may contribute to water reabsorption along the TDL and consequently urinary concentrating ability. To test this hypothesis, we examined water homeostasis in CAII-deficient mice. Animals lacking CAII displayed polyuria and polydipsia that was not driven by a natriuresis or calciuria. Instead, CAII-null mice have more dilute urine than wild-type animals, even after water restriction. This failure to concentrate urine is due to reduced medullary osmolarity, which we suggest is the consequence of reduced water flux through AQP1.

## Materials and methods

### Animal experiments

Generation and genotyping of wild-type and the CAII-deficient mice were performed as described previously (Lewis et al., 1988; Brown et al., 2012). Mice were housed in virus-free conditions and maintained on a 12 h light/dark schedule. Standard pelleted chow [PicoLab Rodent Diet 5053; 20% (wt/wt) protein, 4.5% (wt/wt) fat, 0.81% (wt/wt) calcium, 1.07% (wt/wt) potassium, 0.30% (wt/wt) sodium; and 2.2 IU/g vitamin D_3_] and drinking water was available ad libitum. For all experiments, except where noted, 15 mM sodium citrate was added to the drinking water, as this significantly decreases the mortality of CAII-deficient animals. An equal ratio of both male and female animals between 8 and 14 weeks of age were used. All experiments were performed in compliance with the animal ethics board at the University of Alberta, Health Sciences section, protocol number AUP00000213.

#### Metabolic cage studies

Animals were housed in metabolic cages as previously described for up to 72 h permitting timed collection of urine and measurement of water and chow consumed (Pan et al., 2012; Dimke et al., 2013). At the end of the experiment, the animals were anesthetized with pentobarbital, and blood was collected by retro-orbital venipuncture. Kidneys were next removed and snap frozen in liquid nitrogen as previously (Pan et al., 2012; Dimke et al., 2013) or processed for measurement of tissue osmotic and electrolyte content as described below.

#### Citrate removal studies

Wild-type and CAII-deficient mice were housed in metabolic cages as described above for 24 h with water containing 15 mM sodium citrate. The citrate containing water was then switched to water without sodium citrate and the animals housed in metabolic cages for another 6 days, with urine volume, weight, chow eaten, and water consumed measured every 24 h.

#### Water deprivation study

Wild-type and CAII-deficient mice were housed in metabolic cages for 24 h as described above. After a 24 h urine collection, water was removed and urine collected and weight measured at 3, 7, and 24 h thereafter. Finally, 10 ng of ddAVP (as 0.1 ng/ul ddAVP in saline, Sigma-Aldrich, Oakville, ON, Canada) was injected intraperitoneally and the mice put back in metabolic cages for a further 7 h in order to collect more urine.

### Measurement of serum and urine ${\text{HCO}}_{3}^{-}$, pH, electrolytes, osmolarity, and creatinine

Urine bicarbonate was measured by an in-house developed device as described (Trepiccione et al., 2017). Briefly 20 μl of urine was placed in a hermetically closed chamber. Addition of a strong acid (HCl 0.1 N) converted the bicarbonate present in the urine to CO_2_, and then CO_2_ concentration was determined by a sensor based on non-dispersive infrared-technology (NDIR) (Vaisala CARBOCAP). Finally, the bicarbonate concentration was calculated based on a standard curve of pCO2/[HCO_3_]. Urine pH was assessed by dipstick (Fisher Scientific, Toronto, ON, Canada) as previously (Pan et al., 2012). Urine samples were diluted 1:10 with distilled water and the osmolarity measured with an Advanced Osmometer (model 3D3, Advanced Instruments, Norwood, MA), by freezing point depression. Urine creatinine was determined by the picric acid method as previously using the Creatinine Parameter Assay Kit (R&DSystems Inc., Minneapolis, MN, USA; Pan et al., 2012). Serum creatinine was measured with an Enzymatic Creatinine Test Kit (Diazyme Laboratories, Inc. Poway, CA) according to the manufacturers instructions. Serum sodium and hematocrit were measured with a RAPIDPoint 400/405 System (Siemens Healthcare Diagnostics, Deerfield, IL). Urinary calcium was measured by a colorimetric assay as before (Pan et al., 2012) and urinary sodium concentration determined by atomic flame spectroscopy as described previously (Pan et al., 2012).

### Immunoblotting and quantitative real-time PCR

Aquaporin-2 renal immunoblotting was performed on membrane preparations as previously described (Pan et al., 2012). For all other proteins whole tissue lysate was used. Equal amounts of proteins were loaded and separated by SDS-PAGE and electrotransferred onto Immobilon-P PVDF membranes (Millipore Corporation, MA, USA). Membranes were then rinsed in TBS (in mM: 150 NaCl, 50 Tris-HCl, pH 7.5) and incubated with TBS-TM (TBS containing 0.1% (v/v) Tween-20 and 5% (w/v) skim milk) for 1 h at room temperature. Membranes were then incubated for 16 h at 4°C with either goat anti-AQP1, rabbit anti-CAII, mouse anti-NKCC2 antibodies (DSHB, Iowa, USA), or goat-anti-AQP2 antibody (Santa Cruz, Dallas, TX, USA) at 1:1000 in TBS-TM. After successive washes with TBS and TBS-T (TBS containing 0.1% (v/v) Tween-20), the membranes were incubated with a 1:5000 dilution of the appropriate HRP-conjugated secondary antibody in TBS-TM for 1 h at 20°C and then washed further with TBS-T. Proteins were detected using Western Lightning™ Chemiluminescence Reagent Plus (PerkinElmer Las, Inc., MA, USA), and visualized with a ChemiDoc™ Touch imaging System (Bio-Rad Laboratories, Mississauga, ON, Canada). Quantitative densitometric analyses were performed using Image Lab (Bio-Rad Laboratories, Mississauga, ON, Canada) and beta actin was used as a loading control with all samples normalized to its expression.

Quantitative real time polymerase chain reaction (qPCR) was carried out on whole renal tissue as previously (Pan et al., 2012; Dimke et al., 2013; Rievaj et al., 2013). Total mRNA was isolated from half a kidney using TRIzol Reagent (Invitrogen, Carlsbad, CA, USA) as per the manufacturer's instructions. Isolated total mRNA was then reverse transcribed into cDNA. All primers and probes were from Applied Biosystems Inc. (Foster City, CA, USA). For qPCR, 5 μl (125 ng of cDNA) was used as a template to determine the gene expression of CAII (Cat#: Mm01281788_m1), AQP1 (Cat#: Mm01326466_m1), AQP2 (Cat#: Mm00437575_m1), NKCC2 (Forward: TGCTAATGGAGATGGGATGC, Reverse: CAGGAGAGGCGAATGAAGAG, Probe: TGGAGTTGTGAAGTTTGGATGGGTGA) and 18S ribosomal RNA (Cat#: Mm03928990_g1). A mixture consisting of TaqMan universal qPCR master mix (Applied Biosystems Inc, Foster City, CA, USA), primer, probe, and RNAse free water was prepared and added to the cDNA in a 384-well plate (Applied Biosystems Inc, Foster City, CA, USA). As an internal control mRNA levels of the housekeeping gene 18S ribosomal RNA were determined. Expression levels were quantified with an ABI Prism 7900 HT Sequence Detection System (Applied Biosystems Inc, Foster City, CA, USA). 18S was chosen for normalization of RNA as none of the experimental perturbations resulted in a significant change in its expression.

### Sectioning of tissue for microscopy

Kidneys isolated from wild-type or CAII-deficient mice after water restriction and vasopressin administration were immersion fixed for 3 h in 10% formalin and stored in PBS until paraffin embedding. Embedding was done on a Tissue-Tek Vacuum Infiltration Processor 6 (Sakura Finetek, Torrance, CA, USA). Tissues were sectioned on a HM 355S Automatic Microtome (Thermo Scientific) microtome at 2 μm.

### Immunohistochemistry

Staining of PFA-fixed paraffin-embedded tissue was done as previously described with minor modifications (Alexander et al., 2015; Beggs et al., 2017). Sections were rehydrated in xylene followed by a series of graded ethanol before being subjected to heat-induced antigen retrieval with either 10 mM Tris and 0.5 mM EGTA buffer (pH 9.0). Endogenous peroxidase enzymes and free aldehyde groups were blocked using 0.6% H_2_O_2_ and 50 mM NH_4_Cl in PBS, respectively. Sections were blocked and then incubated overnight at 4°C with primary antibody (1% BSA, 1% skimmed milk, and 0.1% Triton X-100 in PBS). The following antibodies were used. (1) Rabbit polyclonal antibodies against NKCC2 (1:500, Sigma-Aldrich, St Louis, MO), (2) Rabbit polyclonal antibodies against AQP1 (1:300, sc-20810, Santa Cruz). (3) Rabbit polyclonal antibodies against AQP2 have been previously described (1:1000)(Dimke et al., 2007). (4) Phosphorylated-AQP2 (p-AQP2, AN244-pp-AP) an affinity-purified rabbit polyclonal antibody to serine 256 phosphorylated AQP2 has previously been described (1:300)(Christensen et al., 2000). Sections were incubated with horseradish peroxidase-conjugated secondary antibodies (DakoCytomation) and visualized using the Liquid DAB+ Substrate Chromogen System (K3467, DakoCytomation). Finally, sections were counterstained with hematoxylin, dehydrated, and mounted using Aquamount (VWR, Herlev, Denmark). Sections were imaged on a Olympus BX51 microscope and due to morphology, evaluation of membrane localization performed with a 40x lens.

### Measurement of osmolarity, urea, and electrolyte concentration of renal cortex, inner stripe of the outer medulla and inner medulla

Interstitial osmolarity was evaluated as previously described (Trepiccione et al., 2016). Briefly cortex, inner stripe of the outer medulla or inner medulla was dissected from freshly isolated kidneys of either wild-type or CAII-deficient mice. After weighing, the tissue was dried over night then reweighed. The dry tissue was rehydrated in distilled water and then pulverized with an ultrasonic cleaner (Cat# B-220, Branson Cleaning Equipment Company, Shelton, Conn, USA). Samples were then diluted in ddH_2_O and then urea (kit from QuantiChrom, cat# DIUR-100 as per manufactures instructions) osmolarity, or urinary electrolytes were determined.

### Statistical analysis

Data are presented as means ± SEM. Paired or unpaired Student's *t*-tests or analysis of variance were carried out to determine statistical significance as appropriate. Tests were performed using Prism 6.0b software (Graphpad software Inc., La Jolla, CA), and *p*-values < 0.05 were considered statistically significant.

## Results

### CAII-deficient mice are polydipsic and polyuric

**Figure 1:**
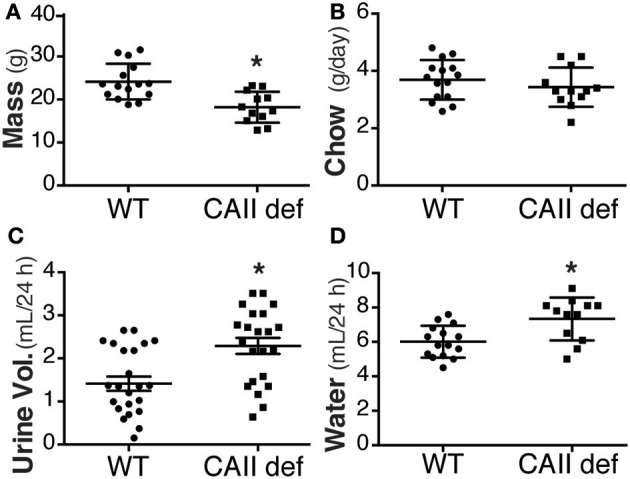
CAII-deficient mice are polydipsic and polyuric. **(A)** Weight **(B)** Chow consumed **(C)** Urine volume in 24 h and **(D)** Water consumed in wild-type (WT) and CAII deficient mice (CAII def). ^*^Represents *p* < 0.05.

In order to asses the potential physiological role of the CAII-AQP1 interaction, we examined water handling in an animal model devoid of CAII. To this end, we employed CAII-deficient mice, which were generated by random mutagenesis (Lewis et al., 1988). These animals lack detectable CAII in all tissue examined, including the kidney (Spicer et al., 1979). Wild-type and CAII-deficient mice were housed in metabolic cages to measure food and water intake and to obtain 24 h urine collections. The CAII-deficient mice weighed less than their wild-type littermates, however, consumed an equivalent amount of chow (Figures 1A,B). Surprisingly they drank significantly more water and produced a greater urine volume than wild-type animals (Figures 1C,D). When urine volume and water intake were normalized to the animal's weight this difference was even more pronounced (water intake 0.27 ± 0.03 vs. 0.38 ± 0.03 mL^*^g^−1*^24 h^−1^ and urine output 0.25 ± 0.03 vs. 0.31 ± 0.03 mL^*^g^−1*^24 h^−1^ wild-type vs. CAII deficient). Consistent with intravascular volume contraction CAII-deficient mice also had an increased hematocrit and hemoglobin level (Table 1).

**Table 1:** Serum analysis and fractional excretion of Na^+^ and Ca^2+^.

	**Wild-Type (*n* = 6)**	**CAII def (*n* = 6)**	***P*-value**
Na^+^ (mmol/L)	149 ± 1	149 ± 1	0.79
Ca^2+^ (mmol/L)	2.58 ± 0.12	2.34 ± 0.09	0.23
Creatinine (μmol/L)	17 ± 3	16 ± 1	0.38
CrCl (μL/min)	96 ± 36	114 ± 23	0.70
FE_Na_	20 ± 2	22 ± 3	0.48
FE_Ca_	8.4 ± 1.8	7.0 ± 1.9	0.88
Hct (%)	34 ± 1	37 ± 1	0.03
Hb (g/dL)	11.6 ± 0.2	12.5 ± 0.4	0.04

*FE, fractional excretion, CrCl, creatinine clearance, Hct, hematocrit and Hb, hemoglobin*.

**Figure 2:**
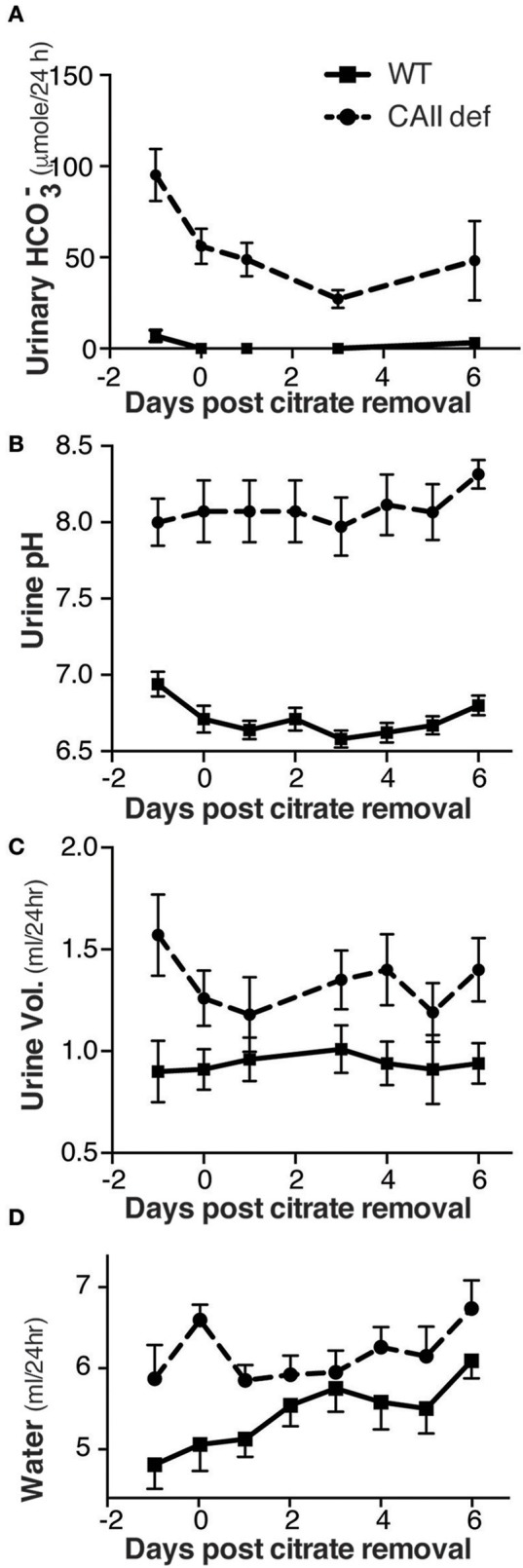
CAII-deficient mice remain polyuric when not fed alkali. **(A)** Urine bicarbonate excretion, **(B)** Urine pH, **(C)** Urine volume and **(D)** Water drank in wild-type and CAII-deficient mice before and for 6 days after removal of citrate from the drinking water.

In order to exclude that the sodium citrate, supplied to all animals in drinking water from weaning, was not contributing to this phenotype, we housed both wild-type and CAII-deficient mice in metabolic cages for 24 h, then removed the alkali from their drinking water and collected urine for 6 days. This resulted in a reduction in both urinary bicarbonate excretion and urinary pH in both genotypes (Figures 2A,B). Consistent with a mixed primary and distal renal tubular acidosis the CAII-deficient mice had bicarbonate wasting and more alkaline urine regardless of being supplemented with alkali. Importantly, the removal of sodium citrate from the drinking water failed to restore the urine volume or amount of water consumed by CAII-deficient mice to that of wild-type animals (Figures 2C,D). CAII-deficient mice are therefore polydipsic and polyuric relative to their wild-type littermates, findings consistent with impaired tubular water reabsorption.

### Polyuria in CAII-deficient mice does not result from natriuresis or hypercalciuria

**Figure 3:**
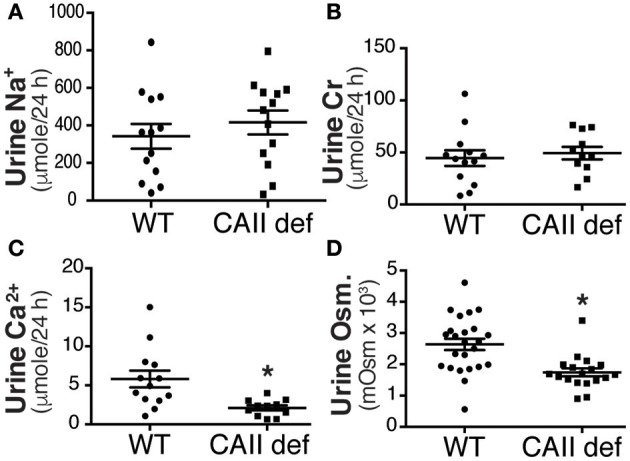
CAII-deficient mice display a dilute urine. **(A)** Urinary sodium excretion, **(B)** Urinary creatinine excretion, **(C)** Urinary calcium excretion and **(D)** Urine osmolarity in wild-type and CAII-deficient mice. ^*^Represents *p* < 0.05.

Carbonic anhydrase inhibitors such as acetazolamide are weak diuretics. A proposed mechanism mediating this diuresis is the inhibition of proximal tubular sodium reabsorption, and a subsequent natriuresis driven osmotic diuresis. In order to examine whether urinary sodium excretion resulted in increased urine volume and polydipsia in CAII-deficient mice we measured urinary sodium excretion (Figure 3A). This was not different between CAII-deficient and wild-type mice nor was the fractional excretion of sodium (Table 1). Impaired proximal tubule sodium chloride reabsorption might decrease glomerular filtration rate via increased delivery to the macula densa inducing tubuloglomerular feedback, thereby complicating the interpretation of these results. We therefore measured urinary creatinine excretion as a surrogate marker of glomerular filtration rate and found it unaltered in the CAII-deficient animals (Figure 3B). Further, we measured plasma creatinine and calculated creatinine clearance. It was unaltered between genotypes (Table 1).

Increased urinary calcium excretion is associated with increased urine volume. We therefore measured urine calcium excretion and found that it was not increased in the CAII deficient mice (Figure 3C), excluding this as a cause of polyuria. Next we examined urine osmolarity and found that the CAII-deficient animals had more dilute urine than wild-type (Figure 3D). This is consistent with a concentration defect causing polyuria in CAII-deficient mice.

### CAII deficient mice have a urinary concentration defect

**Figure 4:**
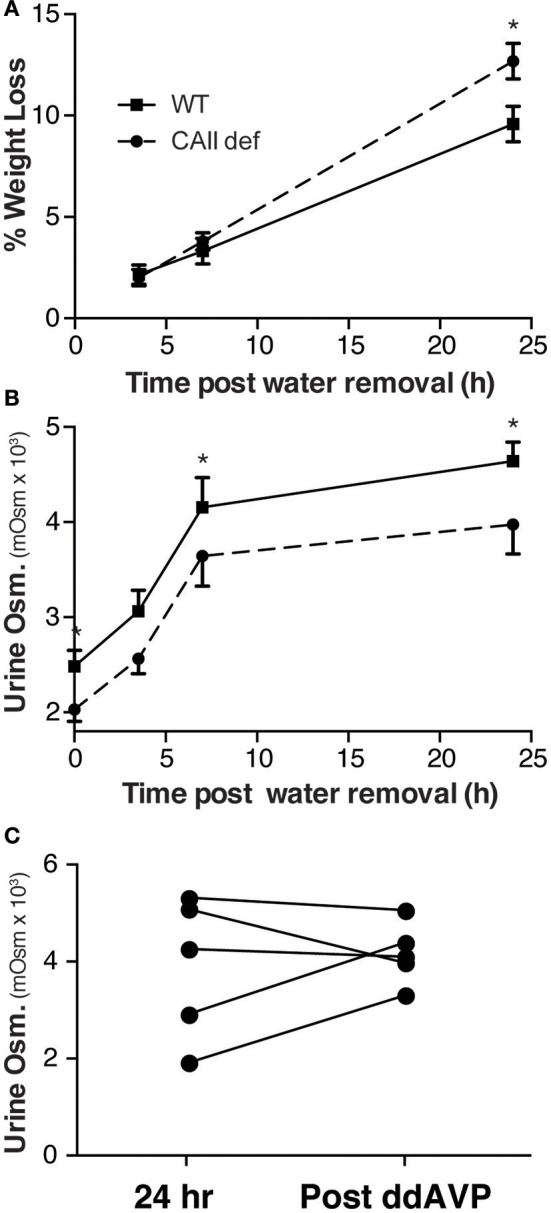
CAII-deficient mice have a urine concentration defect. **(A)** Weight and **(B)** Urine osmolarity post removal of water from the metabolic cage in wild-type and CAII-deficient mice. **(C)** Urine osmoarity before and after ddAVP administration to CAII-deficient mice only. ^*^Represents *p* < 0.05.

In order to assess whether CAII-deficient mice have a urinary concentration defect, we performed a water deprivation study. To this end, mice were housed in metabolic cages for 24 h with water to collect a baseline urine sample and were then water deprived and urine collected over the next 24 h. Consistent with a failure to concentrate their urine, the CAII-deficient animals had lost more weight at the end of the experiment than wild-type mice (Figure 4A). Although the CAII-deficient mice were able to concentrate their urine, their ability to concentrate remained consistently lower than wild-type mice (Figure 4B). After 24 h of water deprivation, we administered ddAVP and collected more urine. CAII-deficient mice failed to further increase their urine concentration after ddAVP administration (Figure 4C), excluding a lack of ADH production from mediating the concentration defect. Thus, CAII-deficient mice have a renal cause for their urinary concentration defect.

### CAII deficient mice have reduced medullary osmolarity

**Figure 5:**
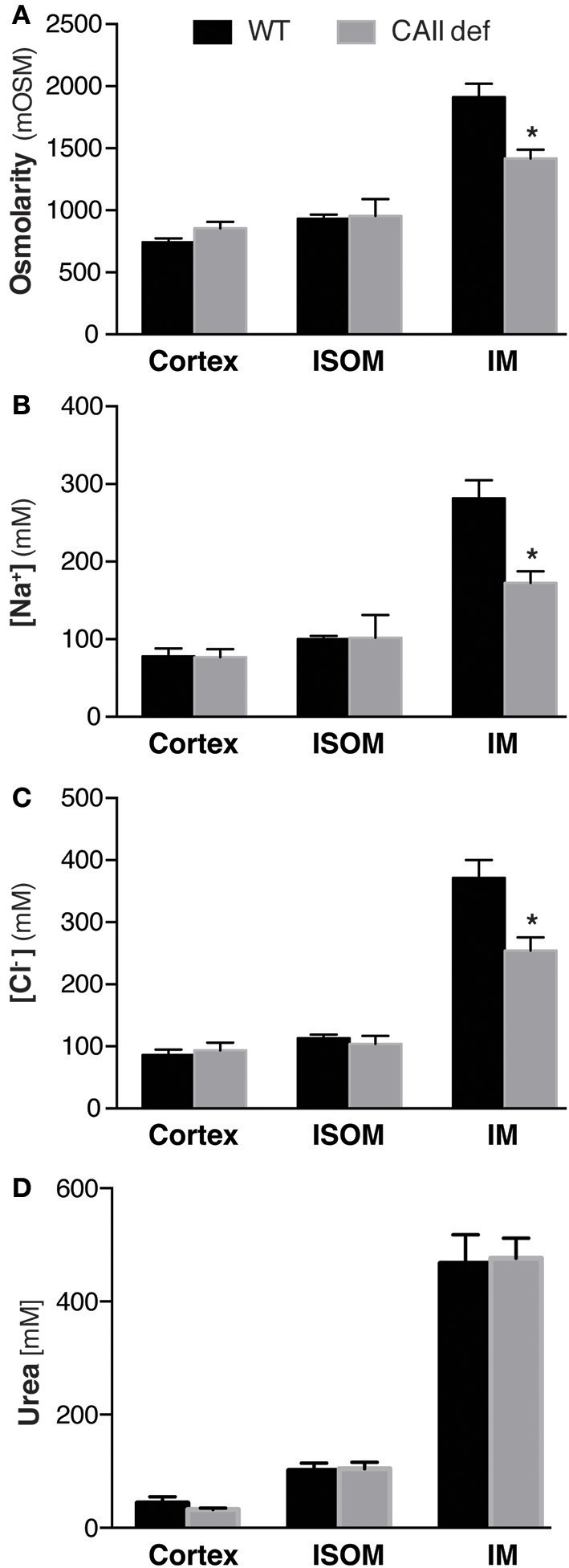
CAII-deficient mice display a dilute medullary interstitium. Renal cortical (Cortex), inner stripe of the outer medulla (ISOM) and inner medulla (IM) **(A)** Osmolarity, **(B)** Sodium concentration, **(C)** Chloride concentration and **(D)** Urea concentration. ^*^Represents *p* < 0.05.

CAII physically interacts with AQP1 to increase water flux through the channel (Vilas et al., 2015). Both CAII and AQP1 are expressed in the TDL where water reabsorption is necessary to concentrate the medullary interstitium. We therefore hypothesized that the CAII-deficient mice may have polyuria and dilute urine as they cannot increase their medullary interstitial osmolarity to the same extent as wild-type mice. To test this hypothesis, we measured the osmolarity of the renal cortex, inner stripe of the outer medulla, and the inner medulla. We found that the CAII-deficient mice had a lower inner medullary osmolarity than wild-type animals, yet the other regions of the kidney were not different (Figure 5A). We also measured the concentration of sodium and chloride and found that it was reduced in the inner medulla but not in the other regions of the kidney (Figures 5B,C). Finally, we measured urea concentration in the different regions of the kidney, and in contrast to sodium and chloride, we found no change in urea concentration in the inner medulla (Figure 5D). Together this data is consistent with reduced water reabsorption in the TDL, leading to a reduced inner medullar concentration and consequently a reduced ability to concentrate the urine.

### CAII deficient mice display unaltered renal expression of AQP2 and NKCC2 but have increased AQP1 expression

**Figure 6:**
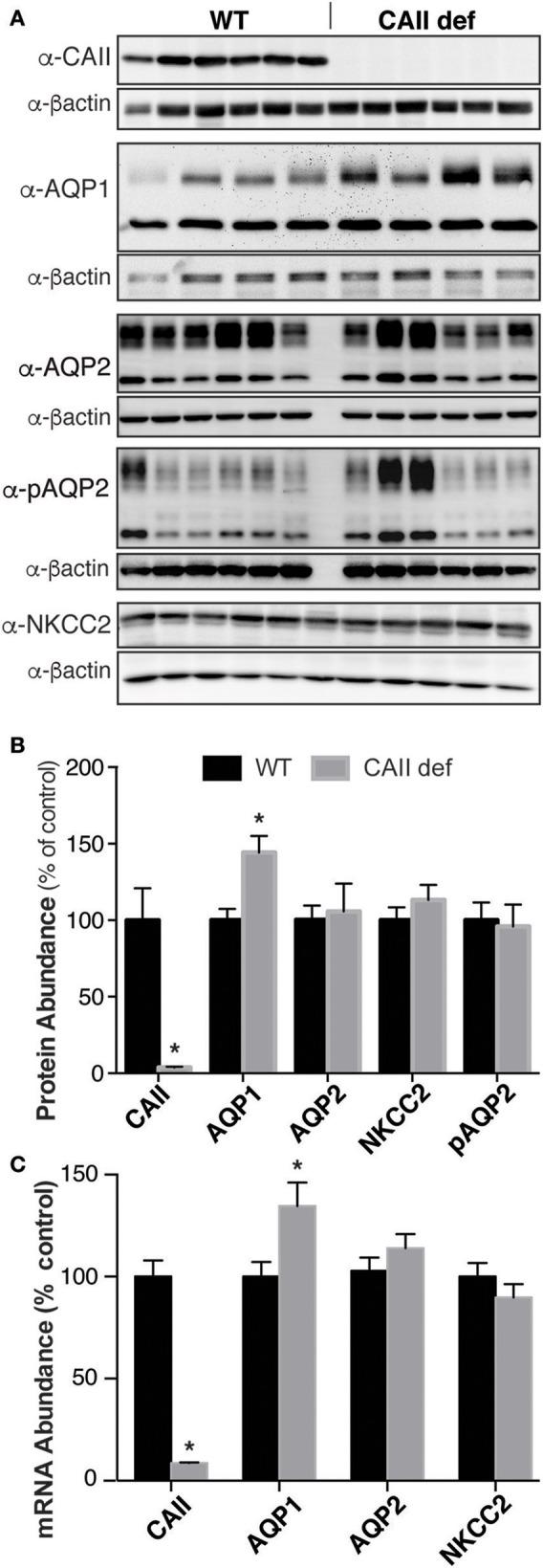
CAII-deficient mice have altered Aquaporin-1 expression. **(A)** Representative immunoblots and **(B)** Protein quantification listed, all samples were normalized to beta actin and the results presented as a % of wild-type. **(C)** mRNA quantification, ^*^Represents *p* < 0.05.

CAII is expressed in the kidney in most tubular segments where it physically and functionally interacts with many transporters and channels, including AQP1 (Schwartz, 2002; Vilas et al., 2013). Given the multiple other transport pathways involved in urine concentration, we sought to examine the localization and expression of the transporters involved in urinary concentration. As expected, we were unable to detect CAII mRNA or protein in the CAII deficient mice (Figures 6A–C). Aquaporin-2 responds to ADH by trafficking into the apical membrane of principal cells, where it permits the movement of water into the interstitium, thereby concentrating the urine (Nielsen et al., 1993a). This is associated with phosphorylation of aquaporin-2 at serine 256 (Christensen et al., 2000). We were unable to detect a difference in expression of either total AQP2 or aquaporin-2 phosphorylated at serine 256 between wild-type and CAII-deficient mice (Figures 6A–C). The sodium chloride cotransporter (NKCC2) is expressed in the apical membrane of the thick ascending limb (Ecelbarger et al., 1996). It mediates the reabsorption of significant sodium and chloride into the medulla, a process necessary for urinary concentration (Takahashi et al., 2000). No difference was detectable in the expression of NKCC2 either (Figure 6A–C). Aquaporin-1 is expressed along the proximal tubule and the TDL where it mediates water reabsorption. Importantly, expression in the TDL is necessary to generate a concentrated inner medulla and therefore to concentrate urine (Chou et al., 1999). Interestingly whole kidney AQP1 protein and mRNA expression was increased (Figures 6A–C). Original immunoblots for Figure 6A are provided in the Supplemental Figure.

**Figure 7:**
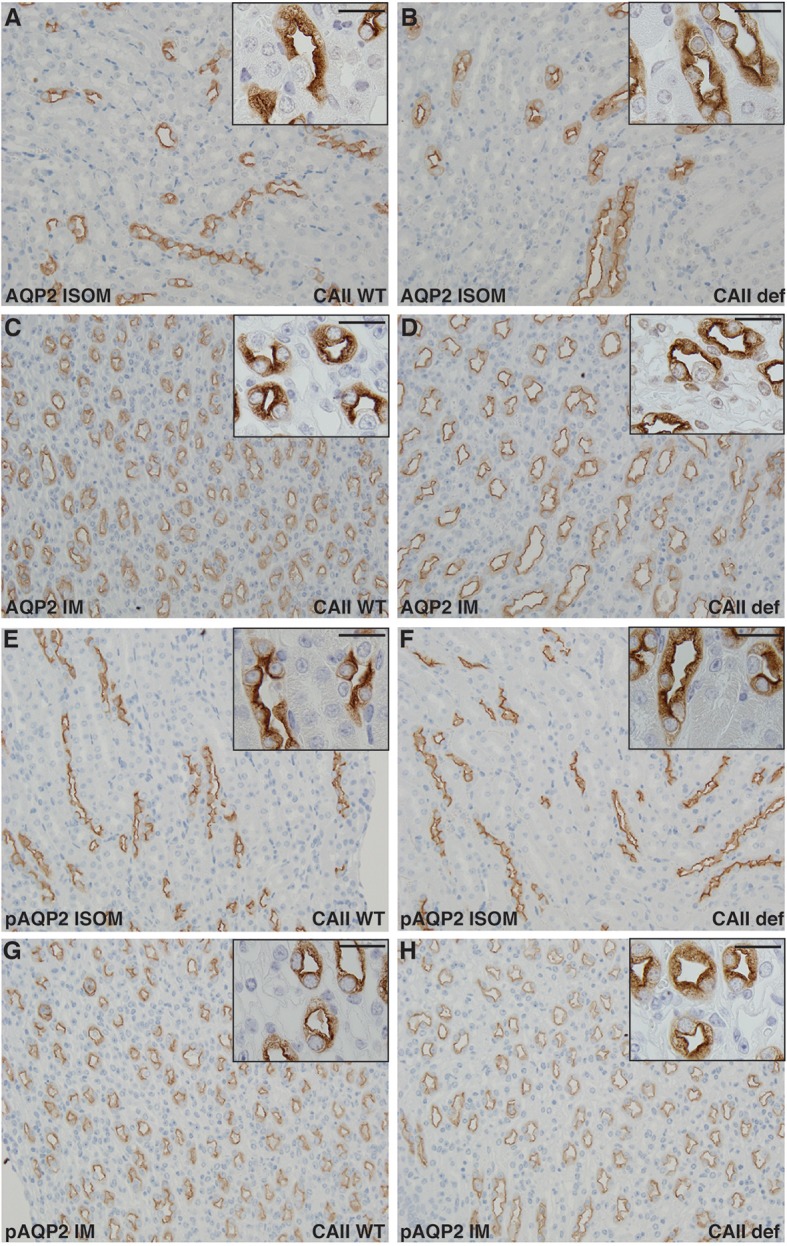
No alterations in Aquaporin-2 localization in CAII deficient mice. Renal sections from wild-type **(A,C,E,G)** or CAII-deficient **(B,D,F,H)** immunostained for aquaporin-2 (AQP2) A-D or serine 256 phosphorylated aquaporin-2 (pAQP2). Representative images from the inner stripe of the outer medulla (ISOM) **(A,B,E,F)** and from the inner medulla (IM) **(C,D,G,H)** are displayed. The scale bar represents 20 μM on the low magnification image and 50 μM on the higher magnification inset image.

Finally, we examined the renal localization of transport proteins involved in urine concentration. To do so, we performed immunohistochemistry on kidney sections from wild-type and CAII-deficient mice after 24 h of water deprivation and ddAVP administration in order to ensure maximal shuttling into the apical membrane of the respective nephron segments. The concentration defect observed in CAII-deficient mice did not appear to result in significant alterations in the localization of AQP2 to apical membrane domains in either the inner stripe of the outer medulla or the inner medulla between groups (Figures 7A–D). Shuttling of AQP2 into the apical plasma membrane of principle cells is associated with phosphorylation of serine 256 (Christensen et al., 2000). We therefore examined the expression of serine 256 phosphorylated AQP2, and again found no difference in localization between wild-type and CAII-deficient mice in either the inner stripe of the outer medulla nor in the inner medulla (Figures 7E–H).

**Figure 8:**
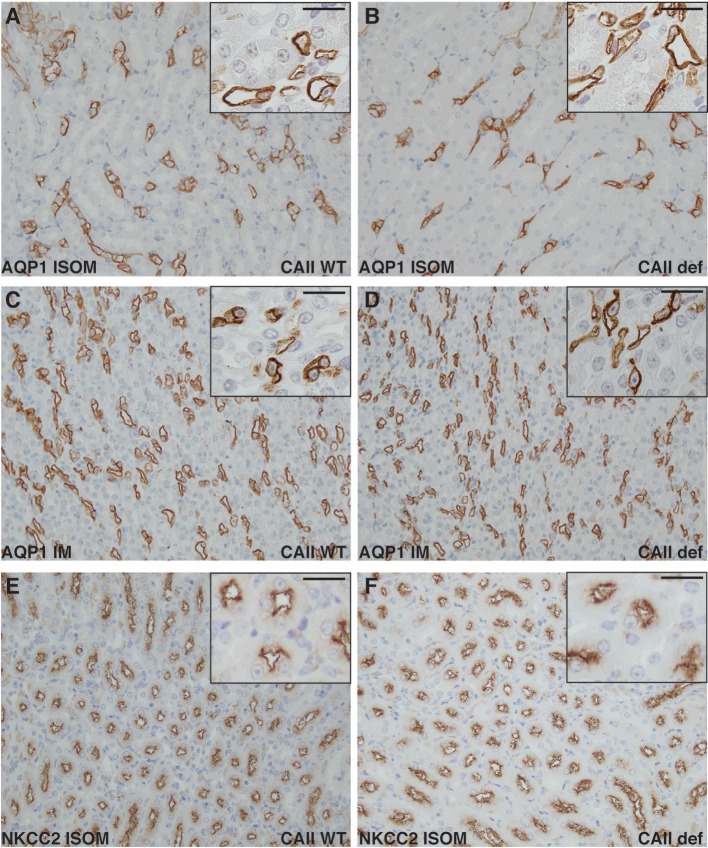
Neither NKCC2 nor aquaporin-1 distribution is altered in CAII deficient mice. Renal sections from wild-type **(A,C,E)** or CAII-deficient mice **(B,D,F)** immunostained for aquaporin-1 (AQP1) **(A–D)** or NKCC2 **(E,F)**. Representative images from the inner stripe of the outer medulla (ISOM) **(A,B,E,F)** and from the inner medulla **(C,D)** are displayed. The scale bar represents 20 μM on the low magnification image and 50 μM on the higher magnification inset image.

Next, we examined the localization of NKCC2 in both wild-type and CAII-deficient mice in the inner stripe of the outer medulla (Figures 8E,F). No marked differences were apparent in localization to apical membrane domains between genotypes. Aquaporin-1 localized to apical membrane domains to a similar extent in both wild-type and CAII-deficient animals in both the inner stripe of the outer medulla and in the inner medulla (Figures 8A–D). Given the appropriate localization of all transporters studied and the phenotype of the CAII-deficient mice, the most likely explanation for polyuria in the null animals is thus reduced water reabsorption from the TDL through AQP1.

## Discussion

We observed that CAII-deficient mice have polyuria and polydipsia. This is not the result of increased urinary calcium or sodium excretion. CAII-deficient animals instead have dilute urine and are not able to concentrate their urine to the same extent as wild-type mice when water deprived. In the absence of altered AQP2 expression or localization, this appears to be the direct result of a failure to increase the medullary osmolarity to the same extent as wild-type mice. The medullary interstitium is concentrated by the counter current multiplier mechanism, which depends on NaCl reabsorption from the TAL via NKCC2 (Ecelbarger et al., 1996), which does not appear altered in CAII-deficient mice. This process is also dependent on the concentration of urea in the medullary interstitium, which also remains unchanged. Finally water reabsorption from the TDL is also required to generate a concentrated medullary interstitium. This occurs through AQP1. Water flux through AQP1 is enhanced via a physical and functional interaction with CAII (Vilas et al., 2015). We therefore propose that in the absence of CAII, water flux through AQP1 is reduced in the TDL, limiting the ability to concentrate the interstitium of the renal medulla. This, in turn, results in the concentration defect observed.

CAII-deficient mice display both a respiratory (Lien and Lai, 1998) and renal tubular acidosis (Lewis et al., 1988). The renal tubular acidosis is both a result of impaired proton secretion in the collecting duct and as shown in Figure 2, a failure to reabsorb bicarbonate. The etiology of the respiratory acidosis is proposed to be due to a defect in the ability of red blood cells to transport carbon dioxide to the lungs (Lien and Lai, 1998). This process is dependent on CAII mediated catalysis of water and carbon dioxide to bicarbonate and a proton in the periphery. The reverse reaction occurs in the lungs, permitting expiration of water, and carbon dioxide. Interestingly the movement of CO_2_ and water into the red blood cell (and its subsequent exit in the lungs) is dependent upon AQP1, whose activity is augmented by CAII (Vilas et al., 2015). It is therefore possible that altered water flux across the red blood cell membrane contributes to the respiratory acidosis observed in CAII deficient mice.

Under conditions of water deprivation, ADH is released from the pituitary gland. This hormone binds the vasopressin type 2 receptor (V2R) in the basolateral membrane of collecting duct principal cells signaling the insertion of AQP2 water channels into the apical membrane (Olesen and Fenton, 2017). In so doing, it permits water to be reabsorbed down its concentration gradient back into the circulation. Mutations in both the V2R and AQP2 itself result in significant polyuria, a disease known as nephrogenic diabetes insipidus (Deen et al., 1994; Fujiwara et al., 1995). We examined the localization and expression of AQP2 in the kidney and found that it was unaltered between wild-type and CAII deficient mice. Moreover, the absence of marked alterations in the distribution of both AQP2 to apical membrane domains and alterations in AQP2 phosphorylated at serine 256 is inconsistent with an alteration in this pathway. Further, we observed increasing urine concentration upon water deprivation that was not further augmented by ddAVP administration to CAII-deficient mice. This supports that ADH is made, secreted and can signal normally in the CAII-deficient animals. Finally, although there likely is low levels of CAII expressed in principal cells, where AQP2 is also expressed, AQP2 lacks the motif we and others have shown binds CAII (Vilas et al., 2015), making it unlikely that CAII contributes to water reabsorption from the collecting duct.

In order for water to be reabsorbed from the collecting duct, it must have a driving force. This is provided by an increased osmolarity of the interstitium in the renal medulla. Urea is a significant solute contributing to the osmolarity of the medullary interstitium. However, medullary urea concentration was not different between wild-type and CAII-deficient mice. Transport of NaCl out of the thick ascending limb of the loop of Henle is also necessary to generate an increased osmolarity in the medullary interstitium. This is highlighted by polyuria and polyhydramnios observed in patients with antenatal Bartter syndrome (Seyberth and Schlingmann, 2011). NaCl transport in the thick ascending limb predominantly occurs via NKCC2-mediated uptake at the apical membrane. Although we observed reduced medullary osmolarity and sodium and chloride concentration in the medulla of the CAII-deficient mice, it is unlikely a defect in NaCl transport in the TAL for three reasons. First, we did not observe altered NKCC2 expression or membrane localization. Second, we did not observe increased urinary excretion of solutes, neither sodium nor calcium which are a typical manifestation of altered NKCC2 transport in the thick ascending limb (Takahashi et al., 2000; Seyberth and Schlingmann, 2011; Figure 3C and Table 1) and thirdly, deficiencies in NKCC2 transport would reduce the osmolality and NaCl concentration in the ISOM, which we did not observe in the CAII-deficient mice (Figures 5A–C).

Examination of renal sections immunostained for NKCC2 and AQP2, as evaluated by immunohistochemistry, revealed no marked alterations between wild-type and CAII-deficient mice. This is consistent with the expression studies. However, to definitively state that there is no difference in sub-cellular localization immuno-gold electron microscopy is necessary. Further, immunohistochemical evaluation studies may not reflect actual water or sodium transport across a given nephron segment. However, despite the absence of immuno-gold electron microscopy and direct measurements of water and sodium transport across these segments, the phenotype of the CAII-deficient mice is not in keeping with alterations in the TAL or collecting duct concentrating mechanisms. Specifically, TAL transport defects are associated with hypercalciuria and NaCl wasting, which is absent in the CAII-deficient mice (Figure 3C and Table 1), and deficiencies in the concentrating segments of the distal nephron produce severe phenotypes, which should not respond to water deprivation or ddAVP (Rojek et al., 2006; Kortenoeven et al., 2013). This is unlike what is observed in the CAII-deficient mice, as these mice respond to water deprivation, yet fail to concentrate the urine to the same level as wild-type mice.

The other component necessary to generate a concentrated inner medulla is the movement of water out of the TDL. This occurs through the AQP1 water channel (Chou et al., 1999). Indeed, animals lacking AQP1 demonstrate urinary concentrating defects similar to what we observe in CAII-deficient mice (Ma et al., 1998). In contrast to other genes required to generate a concentrated medulla, AQP1 expression was altered in CAII-deficient mice. Interestingly AQP1 expression was increased in CAII-deficient mice. Whether this increase was specific to either the proximal tubule or TDL was not readily apparent. However, given reduced inner medullary osmolarity, urinary sodium and chloride concentration, this suggests that AQP1 was not as effective at transporting water into the medullary interstitium in CAII-deficient mice relative to wild-type animals.

Why aquaporin-1 expression is increased in CAII mice is not clear. AQP1 expression is increased by hypertonicity (Umenishi and Schrier, 2003). Given a reduced medullary osmolarity in these animals, this is unlikely the mechanism. Aquaporin-1 expression is induced by corticosteroids (King et al., 1996; Stoenoiu et al., 2003). CAII-deficient mice demonstrate multiple abnormalities, which may increase their corticosteroid level and subsequently increase renal aquaporin-1 expression (Lewis et al., 1988; Lien and Lai, 1998). Alternatively, renin can increase aquaporin-1 expression (Bouley et al., 2009). The CAII-deficient mice have a phenotype consistent with volume contraction, which would increase renin and consequently AQP1 expression. In this latter instance increased aquaporin-1 expression would be a response to decreased AQP1 transport capacity in the absence of CAII, as we have demonstrated that CAII physically and functionally interacts with AQP1 to increase water flux through the channel (Vilas et al., 2015). Thus, the absence of CAII would decrease AQP1 water permeability in the TDL leading to reduce inner medullary osmolarity and a urinary concentrating defect as is evident in CAII-deficient mice. This could cause water loss and volume contraction driving renin release that subsequently increases aquaporin-1 expression. However, we must emphasize this mechanism is purely speculative.

In conclusion, CAII deficient mice display polyuria and polydipsia, which is not due to increased urinary sodium or calcium excretion. Instead, they display a dilute urine and impaired urinary concentrating ability that is a consequence of a failure to effectively concentrate the renal medullary interstitium. Given a lack of an alteration in NKCC2 and AQP2 expression and localization and response to water deprivation and dDAVP administration, we propose that this is a consequence of impaired water reabsorption through AQP1 in the TDL.

## Author contributions

All authors have read and edited the paper for important scientific contributions, below other contributions are listed. DK, FT, and HD: experimental design, data collection, and analysis; WP and MB: data collection and analysis; FT: experimental design, data collection, and analysis; RC and DE: experimental design; RTA: experimental design, data analysis, wrote the first draft, and incorporated all edits from co-authors, is the senior author responsible for the work.

### Conflict of interest statement

The authors declare that the research was conducted in the absence of any commercial or financial relationships that could be construed as a potential conflict of interest.

## References

[B1] Alexander R. T. Beggs M. R. Zamani R. Marcussen N. Frische S. Dimke H. (2015). Ultrastructural and immunohistochemical localization of plasma membrane Ca^2+^-ATPase 4 in Ca^2+^-transporting epithelia. Am. J. Physiol. Renal Physiol. 309, F604–F616. 10.1152/ajprenal.00651.2014 26180241

[B2] Becker H. M. Deitmer J. W. (2007). Carbonic anhydrase II increases the activity of the human electrogenic Na^+^/HCO3- cotransporter. J. Biol. Chem. 282, 13508–13521. 10.1074/jbc.M700066200 17353189

[B3] Beggs M. R. Appel I. Svenningsen P. Skjødt K. Alexander R. T. Dimke H. (2017). Expression of transcellular and paracellular calcium and magnesium transport proteins in renal and intestinal epithelia during lactation. Am. J. Physiol. Renal Physiol. 313, F629–F640. 10.1152/ajprenal.00680.2016 28539338

[B4] Bouley R. Palomino Z. Tang S. S. Nunes P. Kobori H. Lu H. A.. (2009). Angiotensin II and hypertonicity modulate proximal tubular aquaporin 1 expression. Am. J. Physiol. Renal Physiol. 297, F1575–F1586. 10.1152/ajprenal.90762.2008 19776169 PMC2801332

[B5] Brown B. F. Quon A. Dyck J. R. Casey J. R. (2012). Carbonic anhydrase II promotes cardiomyocyte hypertrophy. Can. J. Physiol. Pharmacol. 90, 1599–1610. 10.1139/y2012-142 23210439

[B6] Chou C. L. Knepper M. A. Hoek A. N. Brown D. Yang B. Ma T.. (1999). Reduced water permeability and altered ultrastructure in thin descending limb of Henle in aquaporin-1 null mice. J. Clin. Invest. 103, 491–496. 10.1172/JCI5704 10021457 PMC408109

[B7] Christensen B. M. Zelenina M. Aperia A. Nielsen S. (2000). Localization and regulation of PKA-phosphorylated AQP2 in response to V(2)-receptor agonist/antagonist treatment. Am. J. Physiol. Renal Physiol. 278, F29–F42. 10.1152/ajprenal.2000.278.1.F29 10644653

[B8] Deen P. M. Verdijk M. A. Knoers N. V. Wieringa B. Monnens L. A. van Os C. H.. (1994). Requirement of human renal water channel aquaporin-2 for vasopressin-dependent concentration of urine. Science 264, 92–95. 10.1126/science.8140421 8140421

[B9] Dimke H. Desai P. Borovac J. Lau A. Pan W. Alexander R. T. (2013). Activation of the Ca^2+^-sensing receptor increases renal claudin-14 expression and urinary Ca^2+^ excretion. Am. J. Physiol. Renal Physiol. 304, F761–F769. 10.1152/ajprenal.00263.2012 23283989 PMC4959880

[B10] Dimke H. Flyvbjerg A. Bourgeois S. Thomsen K. Frøkiaer J. Houillier P.. (2007). Acute growth hormone administration induces antidiuretic and antinatriuretic effects and increases phosphorylation of NKCC2. Am. J. Physiol. Renal Physiol. 292, F723–735. 10.1152/ajprenal.00276.2006 17062845

[B11] Ecelbarger C. A. Terris J. Hoyer J. R. Nielsen S. Wade J. B. Knepper M. A. (1996). Localization and regulation of the rat renal Na^+^-K^+^-2Cl- cotransporter, BSC-1. Am. J. Physiol. 271(3 Pt 2), F619–F628. 8853424 10.1152/ajprenal.1996.271.3.F619

[B12] Fujiwara T. M. Morgan K. Bichet D. G. (1995). Molecular biology of diabetes insipidus. Annu. Rev. Med. 46, 331–343. 10.1146/annurev.med.46.1.331 7541187

[B13] King L. S. Nielsen S. Agre P. (1996). Aquaporin-1 water channel protein in lung: ontogeny, steroid-induced expression, and distribution in rat. J. Clin. Invest. 97, 2183–2191. 10.1172/JCI118659 8636397 PMC507297

[B14] Kortenoeven M. L. Pedersen N. B. Miller R. L. Rojek A. Fenton R. A. (2013). Genetic ablation of aquaporin-2 in the mouse connecting tubules results in defective renal water handling. J. Physiol. 591, 2205–2219. 10.1113/jphysiol.2012.250852 23359673 PMC3634529

[B15] Krishnan D. Liu L. Wiebe S. A. Casey J. R. Cordat E. Alexander R. T. (2015). Carbonic anhydrase II binds to and increases the activity of the epithelial sodium-proton exchanger, NHE3. Am. J. Physiol. Renal Physiol. 309, F383–F392. 10.1152/ajprenal.00464.2014 26041446 PMC4959884

[B16] Lewis S. E. Erickson R. P. Barnett L. B. Venta P. J. Tashian R. E. (1988). N-ethyl-N-nitrosourea-induced null mutation at the mouse Car-2 locus: an animal model for human carbonic anhydrase II deficiency syndrome. Proc. Natl. Acad. Sci. U.S.A. 85, 1962–1966. 10.1073/pnas.85.6.1962 3126501 PMC279901

[B17] Lien Y. H. Lai L. W. (1998). Respiratory acidosis in carbonic anhydrase II-deficient mice. Am. J. Physiol. 274(2 Pt 1), L301–L304. 9486217 10.1152/ajplung.1998.274.2.L301

[B18] Ma T. Yang B. Gillespie A. Carlson E. J. Epstein C. J. Verkman A. S. (1998). Severely impaired urinary concentrating ability in transgenic mice lacking aquaporin-1 water channels. J. Biol. Chem. 273, 4296–4299. 10.1074/jbc.273.8.4296 9468475

[B19] Nielsen S. DiGiovanni S. R. Christensen E. I. Knepper M. A. Harris H. W. (1993a). Cellular and subcellular immunolocalization of vasopressin-regulated water channel in rat kidney. Proc. Natl. Acad. Sci. U.S.A. 90, 11663–11667. 10.1073/pnas.90.24.11663 8265605 PMC48044

[B20] Nielsen S. Smith B. L. Christensen E. I. Knepper M. A. Agre P. (1993b). CHIP28 water channels are localized in constitutively water-permeable segments of the nephron. J. Cell Biol. 120, 371–383. 10.1083/jcb.120.2.371 7678419 PMC2119528

[B21] Olesen E. T. Fenton R. A. (2017). Aquaporin-2 membrane targeting: still a conundrum. Am. J. Physiol. Renal Physiol. 312, F744–F747. 10.1152/ajprenal.00010.2017 28179252

[B22] Pan W. Borovac J. Spicer Z. Hoenderop J. G. Bindels R. J. Shull G. E.. (2012). The epithelial sodium/proton exchanger, NHE3, is necessary for renal and intestinal calcium (re)absorption. Am. J. Physiol. Renal Physiol. 302, F943–F956. 10.1152/ajprenal.00504.2010 21937605 PMC3330715

[B23] Purkerson J. M. Schwartz G. J. (2007). The role of carbonic anhydrases in renal physiology. Kidney Int. 71, 103–115. 10.1038/sj.ki.5002020 17164835

[B24] Rievaj J. Pan W. Cordat E. Alexander R. T. (2013). The Na^+^/H^+^ exchanger isoform 3 is required for active paracellular and transcellular Ca^2+^ transport across murine cecum. Am. J. Physiol. Gastrointest. Liver Physiol. 305, G303–G313. 10.1152/ajpgi.00490.2012 23764894 PMC4959879

[B25] Rojek A. Füchtbauer E. M. Kwon T. H. Frøkiaer J. Nielsen S. (2006). Severe urinary concentrating defect in renal collecting duct-selective AQP2 conditional-knockout mice. Proc. Natl. Acad. Sci. U.S.A. 103, 6037–6042. 10.1073/pnas.0511324103 16581908 PMC1421336

[B26] Schwartz G. J. (2002). Physiology and molecular biology of renal carbonic anhydrase. J. Nephrol. 15(Suppl. 5), S61–S74. 12027223

[B27] Seyberth H. W. Schlingmann K. P. (2011). Bartter- and Gitelman-like syndromes: salt-losing tubulopathies with loop or DCT defects. Pediatr. Nephrol. 26, 1789–1802. 10.1007/s00467-011-1871-4 21503667 PMC3163795

[B28] Spicer S. S. Stoward P. J. Tashian R. E. (1979). The immunohistolocalization of carbonic anhydrase in rodent tissues. J. Histochem. Cytochem. 27, 820–831. 10.1177/27.4.109495 109495

[B29] Stoenoiu M. S. Ni J. Verkaeren C. Debaix H. Jonas J. C. Lameire N.. (2003). Corticosteroids induce expression of aquaporin-1 and increase transcellular water transport in rat peritoneum. J. Am. Soc. Nephrol. 14, 555–565. 10.1097/01.ASN.0000053420.37216.9E 12595490

[B30] Takahashi N. Chernavvsky D. R. Gomez R. A. Igarashi P. Gitelman H. J. Smithies O. (2000). Uncompensated polyuria in a mouse model of Bartter's syndrome. Proc. Natl. Acad. Sci. U.S.A. 97, 5434–5439. 10.1073/pnas.090091297 10779555 PMC25846

[B31] Trepiccione F. Gerber S. D. Grahammer F. Lopez-Cayuqueo K. I. Baudrie V. Paunescu T. G.. (2016). Renal Atp6ap2/(Pro)renin receptor is required for normal vacuolar H^+^-ATPase function but not for the renin-angiotensin system. J. Am. Soc. Nephrol. 27, 3320–3330. 10.1681/ASN.2015080915 27044666 PMC5084887

[B32] Trepiccione F. Iena F. M. Catalini L. Carpi F. M. Koed M. Frische S. (2017). Measurement of total CO_2_ in microliter samples of urine and other biological fluids using infrared detection of CO_2_. Pflugers Arch. 469, 1267–1275. 10.1007/s00424-017-1997-8 28585052

[B33] Umenishi F. Schrier R. W. (2003). Hypertonicity-induced aquaporin-1 (AQP1) expression is mediated by the activation of MAPK pathways and hypertonicity-responsive element in the AQP1 gene. J. Biol. Chem. 278, 15765–15770. 10.1074/jbc.M209980200 12600999

[B34] Vilas G. L. Loganathan S. K. Liu J. Riau A. K. Young J. D. Mehta J. S.. (2013). Transmembrane water-flux through SLC4A11: a route defective in genetic corneal diseases. Hum. Mol. Genet. 22, 4579–4590. 10.1093/hmg/ddt307 23813972 PMC3889808

[B35] Vilas G. Krishnan D. Loganathan S. K. Malhotra D. Liu L. Beggs M. R.. (2015). Increased water flux induced by an aquaporin-1/carbonic anhydrase II interaction. Mol. Biol. Cell 26, 1106–1118. 10.1091/mbc.E14-03-0812 25609088 PMC4357510

[B36] Vince J. W. Reithmeier R. A. (1998). Carbonic anhydrase II binds to the carboxyl terminus of human band 3, the erythrocyte C1-/HCO3- exchanger. J. Biol. Chem. 273, 28430–28437. 10.1074/jbc.273.43.28430 9774471

[B37] Vince J. W. Reithmeier R. A. (2000). Identification of the carbonic anhydrase II binding site in the Cl-/HCO3- anion exchanger AE1. Biochemistry 39, 5527–5533. 10.1021/bi992564p 10820026

[B38] Zhai X. Y. Fenton R. A. Andreasen A. Thomsen J. S. Christensen E. I. (2007). Aquaporin-1 is not expressed in descending thin limbs of short-loop nephrons. J. Am. Soc. Nephrol. 18, 2937–2944. 10.1681/ASN.2007010056 17942963

