# Supplementary figures and images for: Deficiency of Carbonic Anhydrase II Results in a Urinary Concentrating Defect
Source: Front Physiol. 2018 Jan 5;8:1108. doi: 10.3389/fphys.2017.01108 (PMC5760551; doi:10.3389/fphys.2017.01108)

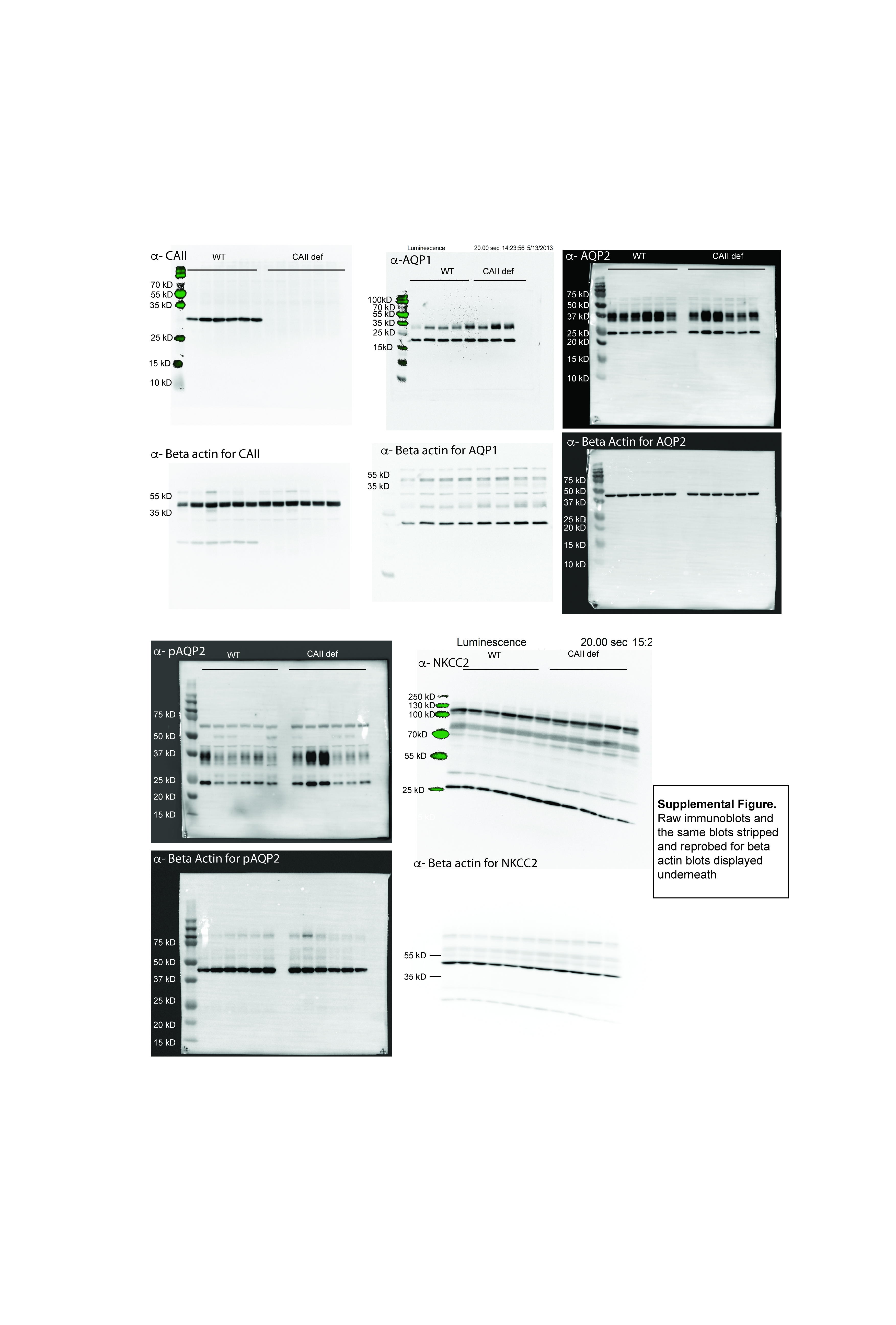

Supplement: Supplementary file 1 [file Image1.JPEG]
